# The Effect of Transversus Abdominis Plane Block on Pain-Related Outcomes in Kidney Transplantation: A Systematic Review with Meta-Analysis and Trial Sequential Analysis

**DOI:** 10.3390/jcm14061879

**Published:** 2025-03-11

**Authors:** Dmitriy Viderman, Mina Aubakirova, Fatima Nabidollayeva, Anuar Aryngazin, Nekane Romero-Garcia, Rafael Badenes, Yerkin G. Abdildin

**Affiliations:** 1 Department of Surgery, School of Medicine, Nazarbayev University, 5/1 Kerey and Zhanibek Khandar Str., Astana 020000, Kazakhstan; drviderman@gmail.com (D.V.); mina.aubakirova@nu.edu.kz (M.A.); 2 Department of Anesthesiology, Intensive Care and Pain Medicine, National Research Oncology Center, 3 Kerey and Zhanibek Khandar, Astana 020000, Kazakhstan; 3 Department of Mechanical and Aerospace Engineering, School of Engineering and Digital Sciences, Nazarbayev University, 53 Kabanbay Batyr Ave., Astana 010000, Kazakhstan; fatima.nabidollayeva@nu.edu.kz (F.N.); anuar.aryngazin@nu.edu.kz (A.A.); yerkin.abdildin@nu.edu.kz (Y.G.A.); 4 Department of Anesthesiology and Surgical-Trauma Intensive Care, Hospital Clínic Universitari, University of Valencia, 46010 Valencia, Spain; nekaneromerog@gmail.com

**Keywords:** anesthesia, donor, kidney transplant, postoperative pain, recipient, renal

## Abstract

**Background/Objectives**: Due to post-surgical discomfort in kidney transplant recipients and donors as well as opioids’ multiple side effects, alternative analgesic methods are required in renal transplant surgeries. This study aimed to evaluate the analgesic effect of the transversus abdominis plane (TAP) block versus no-block controls in kidney transplantation patients. **Methods**: We conducted a meta-analysis with a trial sequential analysis (TSA) of randomized controlled trials (RCTs). We searched for relevant articles in PubMed, Scopus, and the Cochrane Library published before December 2023. Protocol registration: doi.org/10.17605/OSF.IO/PMZJ4. **Results**: A total of 11 RCTs were included in the meta-analysis. The TAP block group had lower pain intensity on postoperative day 1 (mean difference, MD = −0.65 [−0.88, −0.42]; *p* < 0.00001) than the control group. However, the heterogeneity among the included studies was considerable (I^2^ = 93%). Subgroup meta-analysis and TSA revealed a significant pain reduction at 24 h postoperatively in donors (MD = −0.70 [−1.16, −0.24]; *p* = 0.003); heterogeneity was substantial (I^2^ = 67%). The TAP block group also had lower overall morphine consumption within 24 h (MD = −4.82 [−7.87, −1.77]; *p* = 0.002) and cumulative 24 h morphine use (MD = −14.13 [−23.64, −4.63]; *p* = 0.004); however, heterogeneity was considerable (I^2^ = 98% in both cases). The time to first analgesia (hours) was significantly longer in the TAP block group (MD = 5.92 h [3.63, 8.22]; *p* < 0.00001, *n* = 3). There was no significant difference between the groups in postoperative nausea and vomiting (risk ratio, RR = 0.91 [0.49, 1.71]; *p* = 0.78). **Conclusions**: TAP block can lower pain intensity and reduce morphine consumption on the first postoperative day in patients undergoing renal transplantation. Pain reduction is especially notable in the subgroup of donors, but the benefits reported are minimum and certainly not clinically relevant. Larger, well-powered RCTs are warranted to confirm these results and evaluate the effect of TAP block in the subgroup of recipients.

## 1. Introduction

Although there are a few analgesic choices after a kidney transplant operation, there may be significant postoperative discomfort [1]. While effective results are influenced by appropriate postoperative pain management, choices for efficient postoperative analgesia following kidney transplantation have been restricted due to the recipient’s unique features, such as the danger of exacerbating kidney transplant complications, altered renal medication clearance, and the danger of transplanted kidney damage. Currently, opioid intravenous patient-controlled analgesia is the accepted method for pain management following kidney transplantation. Opioids, however, have a number of adverse effects that might increase the risk of infection following kidney transplantation, such as vomiting, nausea, respiratory depression, and weakened immunological response [2].

Following abdominal procedures, a number of regional anesthetic treatments, including epidural analgesia, intercostal, ilio-hypogastric, ilioinguinal, transversus abdominis plane (TAP) blocks, and rectus sheath, are being utilized to provide postoperative analgesia [3]. Analgesia for surgeries affecting the abdominal wall is provided by TAP block, which blocks the first lumbar nerves as they pass through the neurofascial plane between the internal oblique muscles and transversus abdominis, and lower six thoracic nerves [4]. TAP block was initially defined as a landmark-guided procedure that involved inserting a needle at the Petit triangle. Recently, ultrasound-guided techniques of TAP block have been described due to the difficulties of the landmark procedure, which include anatomical variations in the Petit triangle, obese individuals’ challenging palpation of the angle, side effects such as colonic and liver injury, and uncertain local anesthetic dissemination [5].

After abdominal procedures like appendectomy, cesarean section, and hysterectomy, TAP blocks have been effective in lowering postoperative discomfort and the total need for morphine. However, a number of recent trials in patients having cesarean sections have revealed no advantage in the administration of a TAP block, raising doubts about the technique’s applicability to other lower abdominal surgeries [6].

The aim of this systematic review and meta-analysis (SR&MA) was to examine the efficacy of TAP blocks in kidney transplantation.

## 2. Materials and Methods

### 2.1. Protocol

We devised a protocol for the study with the inclusion/exclusion criteria for relevant articles (Protocol registration: doi.org/10.17605/OSF.IO/PMZJ4; 3 December 2023). Trial sequential analysis was conducted to deeper analyze the results after the initial publication of the protocol. All the authors agreed with the devised protocol, further amendments, and the methods. We searched for randomized controlled trials (RCTs) published in the English language that examined analgesic properties of TAP block in kidney transplantation. We used the “Preferred Reporting Items for Systematic Reviews and Meta-Analyses (PRISMA)” [7].

Two authors independently conducted the search for and the screening of RCTs in PubMed, Scopus, and the Cochrane Library published before December 2023. Disagreements were handled by engaging a third author. The search was performed using the following terms and their combinations: “transversus abdominis plane block”, “pain”, “postoperative pain management”, “pain management”, “postoperative pain”, “kidney transplantation”, “kidney transplant surgery”, “renal transplantation”, and “renal transplant surgery”.

### 2.2. Inclusion and Exclusion Criteria

#### 2.2.1. Inclusion Criteria

Patients: adult kidney transplantation patients;

Intervention: TAP block;

Controls: control group;

Outcomes: primary—the intensity of post-surgical pain; secondary—PO opioid requirements, time to first analgesia, and incidents of nausea and vomiting;

Study design: randomized controlled trials (RCTs) published in English;

Publication: from inception to December 2023.

#### 2.2.2. Exclusion Criteria

Patients: pediatric patients;

Intervention: other types of blocks;

Controls: no control groups, other blocks;

Study design: non-RCTs;

Publication: failure to access or obtain the full text.

### 2.3. Data Extraction and Analysis

Two authors independently conducted data extraction. Disagreements were resolved by involving another author. Descriptive data about each study (country, study design, goals, patient age, sample size, local anesthetic volume and concentration, and postoperative analgesia) were extracted into a data table. Numeric data were extracted into an Excel table for further statistical analysis. Missing statistics (mean and standard deviation) were estimated using existing estimation techniques [8,9]. Meta-analysis was performed on the outcomes of 24 h pain intensity, pain scores at 24 h for the subgroups of kidney donors and recipients, 24 h morphine requirements, cumulative morphine requirements at 24 h, time to first analgesic request, and incidence of nausea and vomiting. A random-effects model was used due to the expected heterogeneity. Mean difference was used for continuous variables, and risk ratio was used for dichotomous outcomes. Forest plots were built for each outcome. Statistical significance was set at *p* < 0.05. We assessed heterogeneity by the I^2^ statistic. Subgroup analysis was performed whenever possible. Sensitivity analysis was conducted by observing the change in the effect size when eliminating each study one by one. Trial sequential analysis (TSA) was performed on the outcomes of pain scores at 24 h for the subgroup of donors and recipients, and cumulative morphine requirements at 24 h. Statistical analysis was performed using “Review Manager (RevMan) [computer program]. Version 5.4. The Cochrane Collaboration, 2020” and “Trial Sequential Analysis Viewer (TSA Viewer) [Computer program]. Version 0.9.5.10 Beta. Copenhagen: Copenhagen Trial Unit, Centre for Clinical Intervention Research, Rigshospitalet, 2016.” [10].

### 2.4. Quality Assessment

Each study was examined using the Cochrane risk of bias assessment tool 2 [11]. The studies were ranked as having “low risk”, “some concerns”, or “high risk” of bias. Furthermore, each outcome was evaluated for risk of bias, imprecision, indirectness, and inconsistency using GRADE [12].

## 3. Results

### 3.1. Article Search Results

**Figure 1:**
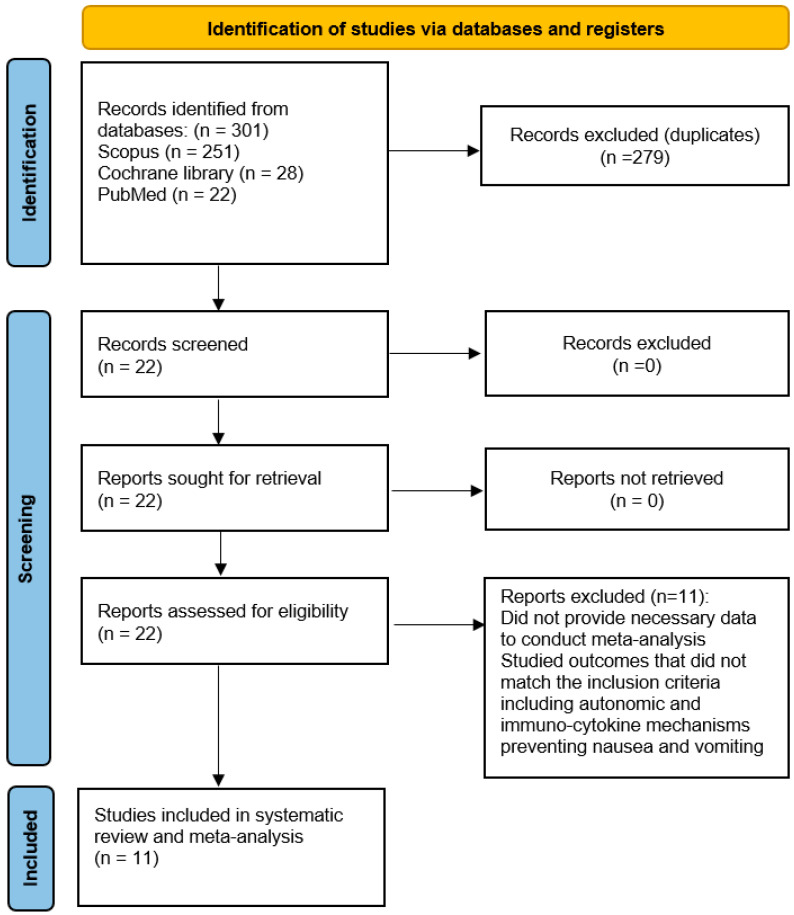
PRISMA flowchart: the study selection process.

We initially identified 301 articles that conformed to the search criteria, but 279 articles were excluded due to duplications, so 22 articles were assessed for eligibility. Eleven articles with 547 patients (273 in the TAP block group and 274 in the control group) were ultimately included in the meta-analysis (Figure 1 and Table 1).

### 3.2. Pain Intensity on Postoperative Day 1

**Figure 2:**
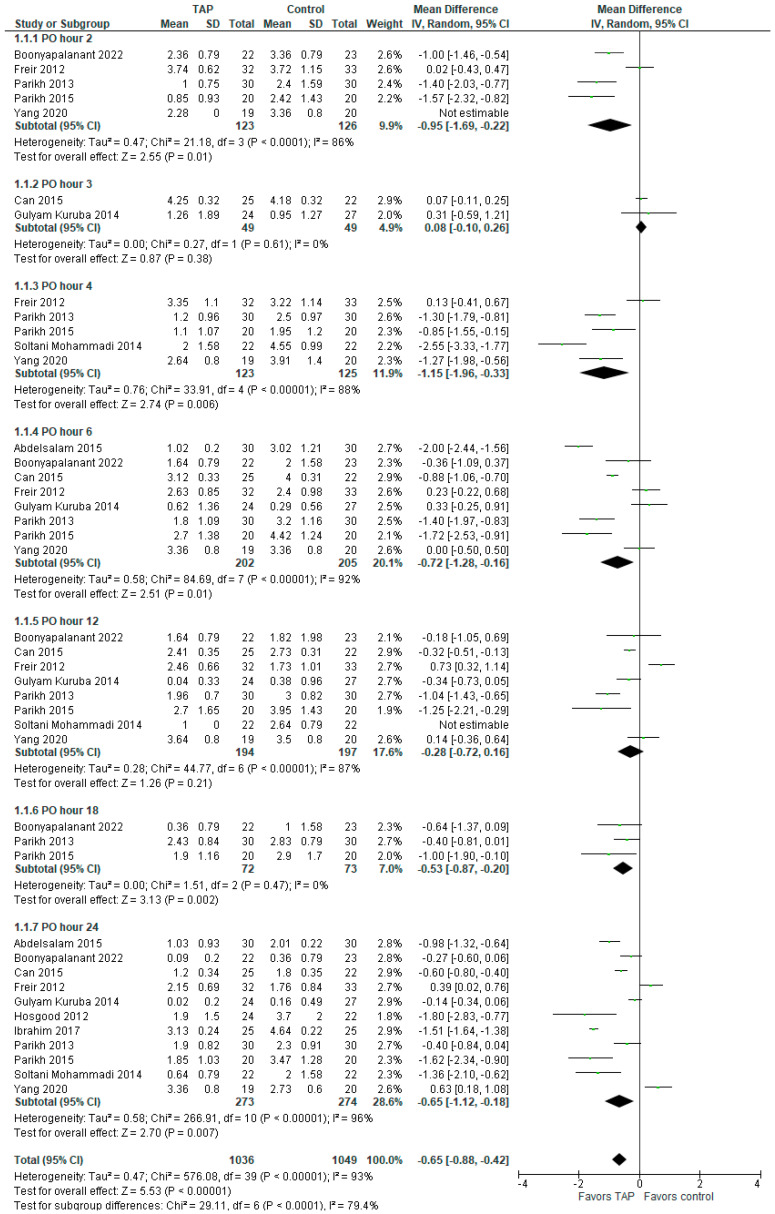
Postoperative day 1 pain intensity score. The forest plot shows pain intensity during the first 24 h post-surgery [1,2,3,4,5,6,13,14,15,16,17].

There was a significant difference between the groups in pain intensity (MD with 95% CI is −0.65 [−0.88, −0.42]; *p* < 0.00001) on postoperative day one (POD 1) in favor of TAP block, but the heterogeneity among the included studies was considerable (I^2^ = 93%). The result is not sensitive to exclusion of any study. The subgroup analysis showed lower pain intensity at PO hours 2, 4, 6, 18, and 24, whereas at PO hours 3 and 12, there were no significant differences between the groups. The forest plot is presented in Figure 2. It should be noted that standard deviation (SD) values were not provided in Freir (2012) [4], so they were taken as the mean of the group SD (e.g., the mean SD of the 10 studies in the ‘PO 24 hour’ subgroup of the TAP block was 0.69, so the SD for Freir (2012) [4] was set at 0.69).

### 3.3. Morphine Requirements Within 24 h

**Figure 3:**
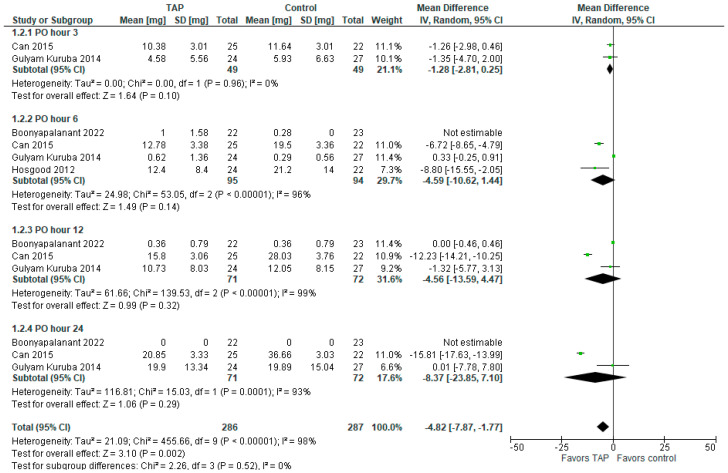
Morphine requirements. The forest plot shows morphine requirements within the first 24 h post-surgery [1,5,6,14].

The forest plot for POD 1 morphine consumption is presented in Figure 3. The model favors the TAP block group over the control group (MD with 95% CI is −4.82 [−7.87, −1.77]; *p* = 0.002) and the result is statistically significant. However, this result is sensitive to exclusion of a study by Can et al. (2015) [5]. Heterogeneity is considerable (I^2^ = 98%). The subgroup analysis does not show significant difference between the groups at PO hours 3, 6, 12, or 24.

### 3.4. Cumulative Morphine Requirements in 24 h

**Figure 4:**
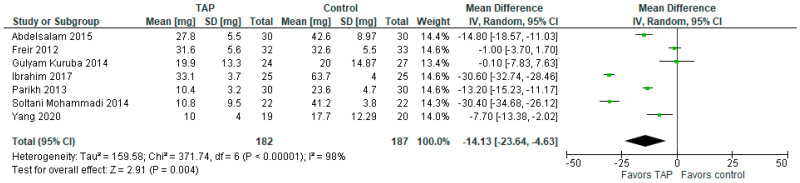
Cumulative morphine requirements in 24 h. The forest plot shows cumulative morphine requirements at 24 h post-surgery [1,2,3,4,13,15,16].

In terms of the cumulative morphine requirements (mg) in 24 h, the model (Figure 4) favors the TAP block group over the control group (MD with 95% CI is −14.13 [−23.64, −4.63]; *p* = 0.004). Heterogeneity is considerable (I^2^ = 98%). This result is statistically significant and not sensitive to exclusion of any study. It should be noted that the values in Ibrahim (2017) [15] were reported in μg fentanyl, whereas in Parikh (2013) [16] they were reported in mg tramadol, so the values in both studies were converted to mg morphine by multiplying the values by 0.1.

### 3.5. Time to First Analgesia (Hours)

**Figure 5:**
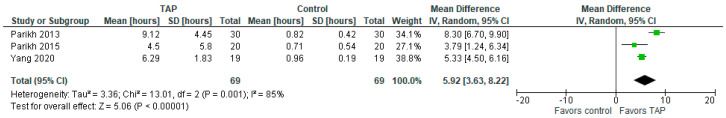
Time to first analgesia. The forest plot shows the time (hours) until the first request for analgesia [2,16,17].

The model (Figure 5) favors the TAP block group over the control group as the time to first analgesia is significantly longer in the former group (MD is 5.92 h and the 95% CI is [3.63, 8.22]; *p* < 0.00001). Heterogeneity is substantial (I^2^ = 85%).

### 3.6. Incidence of Nausea and Vomiting Within 24 h After Surgery

**Figure 6:**
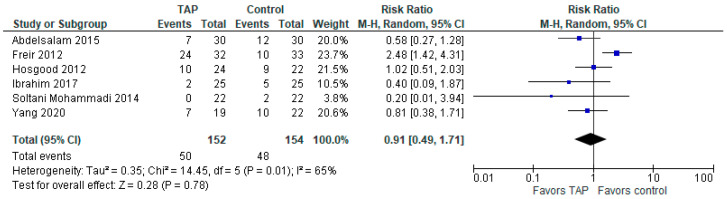
Postoperative nausea and vomiting. The forest plot shows the risk ratio of nausea and vomiting 24 h post-surgery [2,3,4,6,13,15].

The model (Figure 6) shows no difference between the groups (risk ratio with 95% CI is 0.91 [0.49, 1.71]; *p* = 0.78). Heterogeneity is substantial (I^2^ = 65%). It should be noted that one study (Soltani Mohammadi (2014), [3]) only reported the incidence of nausea, but we still included it in the postoperative nausea and vomiting (PONV) analysis, assuming that there were no cases of vomiting in that study.

### 3.7. Subgroup Analysis on Pain Intensity Between Donors and Recipients at 24 h

**Figure 7:**
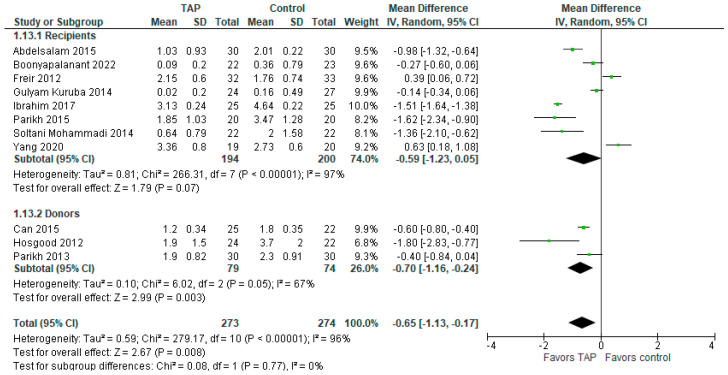
Subgroup analysis on pain intensity at 24 h PO. The forest plot shows the mean difference in pain scores at 24 h post-surgery for the subgroups of donors and recipients [1,2,3,4,5,6,13,14,15,16,17].

The subgroup analysis between recipients and donors on pain intensity at PO hour 24 (Figure 7) shows that in the subgroup of donors, the patients in the TAP block group have significantly less pain intensity than the patients in the control group (MD with 95% CI is −0.70 [−1.16, −0.24]; *p* = 0.003). However, the result is sensitive to the exclusion of either the study of Can (2015) [5] or that of Parikh (2013) [16]. Heterogeneity is substantial (I^2^ = 67%). In the subgroup of recipients, the model does not show such statistically significant difference, but the result is sensitive to the exclusion of either the study of Freir (2012) [4] or that of Yang (2020) [2], in which case the model will favor the TAP block group over the control. The overall result of the model favors the TAP block group over the control group, and the result is not sensitive to the exclusion of any study.

### 3.8. TSA of Pain Intensity at 24 h Postoperatively in Recipients and Donors (Separately/Together)

**Figure 8:**
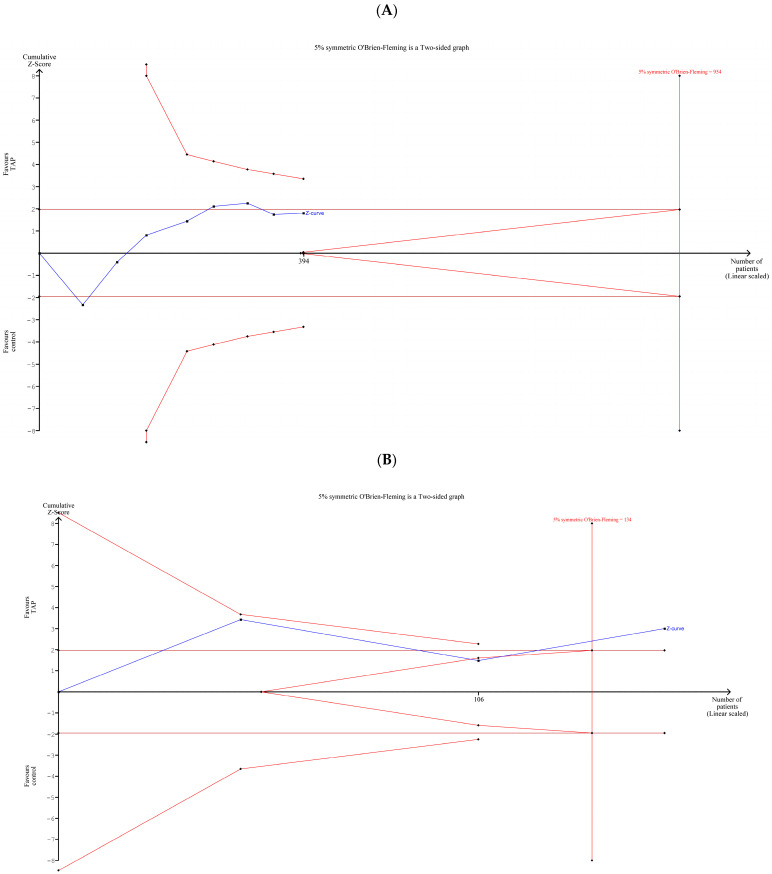

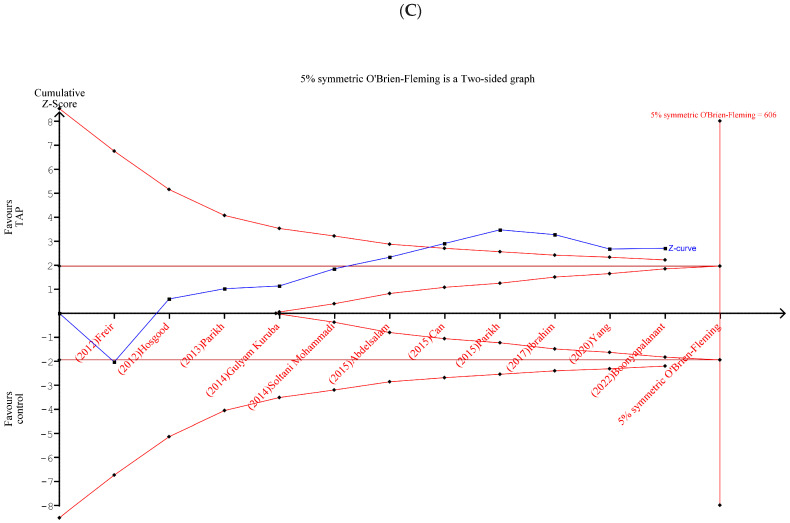
TSA of pain intensity at PO hour 24: (**A**) recipients; (**B**) donors; (**C**) recipients and donors together. Figure 8 shows the results of a trial sequential analysis of pain 24 h after surgery for the recipient and donor groups, both separately and together [1,2,3,4,5,6,13,14,15,16,17].

The TSA of pain intensity at PO hour 24 in the group of only recipients (Figure 8A) shows no statistically significant difference between the TAP block group and the control group. Moreover, Figure 8A shows that there is room for new studies involving up to 560 additional patients (954-394) that would be beneficial for further examining the topic. Both the forest plot in Figure 7 and the TSA plot in Figure 8A show that additional RCTs with the subset of recipients should be conducted to clarify the results on pain intensity on POD1.

In the group of only donors (Figure 8B), the Z-curve (blue line) crossed the curved monitoring boundary before reaching the information size (*n* = 134), so we can conclude that the patients in the TAP block group had significantly less pain intensity than the patients in the control group. This result is consistent with the result for donors in the meta-analysis (Figure 7).

In the group of recipients and donors together (Figure 8C), the Z-curve crossed the curved monitoring boundary before reaching the information size (vertical line), which indicates that the patients in the TAP block group had significantly less pain intensity than the patients in the control group. This result confirms the overall result of the meta-analysis in the forest plot in Figure 7.

### 3.9. TSA of Cumulative Morphine Requirements at 24 h (Only Recipients)

**Figure 9:**
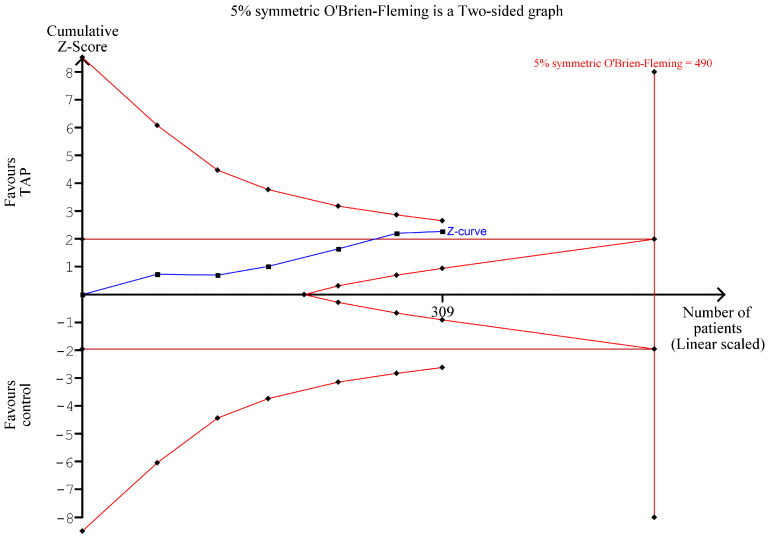
TSA of cumulative morphine requirements on POD1 (only recipients). Figure 9 shows the results of a trial sequential analysis of cumulative morphine requirements 24 h after surgery [1,2,3,4,13,15].

TSA (Figure 9) confirms the results of the meta-analysis (Figure 4), showing a reduction in cumulative morphine requirements at PO hour 24 in the TAP block group. However, the reduction is not statistically significant in terms of TSA, and more studies with up to 181 patients are needed to have a conclusive result. This TSA is based on six studies (all for recipients) [1,2,3,4,13,15]. None of the donor studies (except Parikh (2013) [16]) provided data for this outcome.

### 3.10. Assessment of Methodological Quality

Eight studies had “low risk” of bias based on the Cochrane risk of bias tool, while three had “some concerns” (Table 2). Of the outcomes studied, pain intensity during the first PO 24 h and time to first analgesia (hours) had a low certainty of evidence due to inconsistency, while cumulative morphine requirements, PONV, had a very low certainty of evidence due to inconsistency and imprecision (Table 3).

## 4. Discussion

In this meta-analysis, we observed a statistically significant and not clinically relevant difference between the TAP block group and controls in post-surgical pain scores, morphine requirements within 24 h, and cumulative 24 h morphine consumption. However, the heterogeneity among the included studies was considerable for both pain intensity and opioid consumption (I^2^ = 93% and I^2^ = 98%, respectively), indicating differences between studies regarding the magnitude of benefit of TAP block on these outcomes. There was a statistical and not clinically relevant difference in pain scores at 24 h in the donor but not the recipient subgroup. The TAP block group had a statistically longer time to first analgesic request. We observed no difference between the two arms in terms of PONV.

While postoperative pain and PONV are undesirable side effects of any surgical intervention, morphine consumption is of special importance following kidney surgeries. Morphine is metabolized in the liver into morphine-3- and morphine-6-glucuronide (M3G and M6G). The latter is the major contributor to the drug’s analgesic effect. These metabolism products are then cleared by the kidneys. However, in renal-compromised patients, the metabolites accumulate due to improper creatinine clearance and the inadequate function of the kidneys. Although this accumulation may enhance the analgesic effect following the administration of several doses, it also aggravates adverse effects [13]. Therefore, the careful management of morphine use in renal patients is vital.

In our study, there was a trend towards lower morphine consumption at each individual time point, with a statistically significant overall decrease in the use of opioids within 24 h. Moreover, there was a significant and clinically meaningful difference of −14.13 [−23.64, −4.63] mg between the two groups in cumulative opioid consumption at 24 h. Although TSA did not confirm the statistically significant difference between the two groups, it identified room for future studies to influence the results of this analysis. Therefore, more RCTs on the topic are required to add knowledge to the issue.

Previous studies have demonstrated that TAP block may be an effective way to reduce opioid consumption in renal transplant surgery. A meta-analysis that also included non-randomized and retrospective studies found post- and peri-operative morphine consumption to be lower in the TAP block group following kidney transplantation [14]. Additionally, similarly to our results, their study demonstrated lower postoperative pain intensity within 24 h post-surgery in the TAP block group. However, in contrast to our results, the researchers found that the odds of postoperative nausea and vomiting were increased in the group that did not receive TAP block.

Our results revealed the limited pain-sparing effect of TAP at 2, 4, 6, 18, and 24 h following the surgery. A recent study compared the effect of TAP block and pre-closure local infiltration in kidney recipients [15]. Both groups received 20 mL bupivacaine 0.25%. TAP block was performed using the inside-out technique to lower the risk of visceral organ injury by providing superior visualization. The study found a lower pain intensity at two hours post-operation in the TAP block group. However, beyond this time, the two groups had comparable pain scores and morphine use.

An interesting finding of our study is that TAP block had a pain-sparing effect in kidney donors than recipients. This may be explained by the difference in the surgical approach and the extent of tissue trauma between donors and recipients. Thus, all the included donor surgeries were laparoscopic, while those for recipients included open procedures. Second, donor surgeries are typically performed on the left kidney due to anatomical advantages such as the longer renal vein, easier surgical access, and a longer ureter. Among the included studies that reported the side of the operation, the vast majority of donor surgeries were performed on the left kidney, and the opposite was true for the recipient procedures. Finally, TAP block might be more effective in kidney donors due to potential differences in pain perception and tolerance, as recipients may have pre-existing pain from kidney disease or comorbidities, while donors typically undergo less extensive surgical procedures and may have a lower baseline level of pain. It should be mentioned, however, that in the recipients, the difference in pain scores between the TAP block group and controls was close to being statistically significant, as evident from the graphs. The meta-analysis yielded a difference of −0.59 [−1.23; 0.05] (*p* = 0.07) on a 10-point scale, and the result was sensitive to the exclusion of two studies. The TSA graph also visually represents the proximity of the Z-curve to a statistically significant difference.

Local analgesia, including TAP block, is often used as part of the Enhanced Recovery Pathway (ERP) for kidney transplantation. Still not a widely accepted practice for this procedure, it often involves, among other components of the treatment protocol, educating patients, dietary changes, multimodal analgesia, and opioid-sparing techniques. As such, in a retrospective cohort study, one group of renal transplant patients received US-guided TAP block (30 mL bupivacaine 0.5%) as part of ERP, and postoperative outcomes were compared to controls [18]. The results showed that postoperative pain on the day of operation and on the third day was lower in the intervention group, as was opioid consumption on days zero through three. The main outcome was the length of hospitalization, which also decreased for the TAP block group. Similarly, another study administered TAP block (levobupivacaine 20 mg three times daily for two days) as part of their ERP protocol and compared the results with those of donors and recipients prior to the adoption of the program [19]. They found lower pain scores and a shorter duration of stay at the hospital in the TAP block group compared to those who received PCA only. Therefore, TAP block can be combined with other techniques to reduce the burden of postoperative complications following renal transplant surgery.

One of the main limitations of this meta-analysis is that the included RCTs were relatively small in the number of involved patients. Moreover, as evidenced by the I^2^ statistic, the studies were heterogeneous. This may be the result of the different TAP techniques and analgesics used. Therefore, larger RCTs with similar designs should be conducted to deepen the understanding of the effect of TAP block in kidney donors and recipients.

As the evidence supporting the use of regional anesthesia, particularly interfascial plane blocks in abdominal surgeries such as transplant surgery, continues to expand [20,21,22,23,24,25,26,27,28], it is imperative to compare the impact of different techniques on postoperative short- and long-term outcomes, such as postoperative complications, transplant function, and patient survival. Furthermore, it is crucial to improve the registration of complications associated with regional anesthesia. Despite improvements in clinical practices, local anesthetic systemic toxicity (LAST), associated with the increased volume and dose of local anesthetic required to achieve the desirable effect, remains a significant challenge. This is partly attributed to the growing adoption of ultrasound guidance and diverse interfascial block approaches [29]. LAST onset now tends to be more delayed, with an increasing number of clinical reports linked to fascial plane blocks. Moreover, there is a concerning trend of cases involving non-anesthetist providers. The changing clinical landscape poses a diagnostic challenge, emphasizing the need for educating all healthcare professionals, including physicians, nurses, and allied health professionals, to deepen their understanding about these evolving patterns and associated risks [30,31,32].

Given TAP block’s potential to reduce pain intensity and opioid consumption, integrating this regional anesthesia technique into clinical practice would be beneficial [33,34]. However, due to risks associated with interfascial blocks, established recommendations should be adopted in clinical practice [33,34,35,36,37,38]. Surgeons, anesthesiologists, and nurses must have a thorough understanding of LAST and should be adequately trained to promptly identify and address it. It is also necessary to maintain effective communication among all the members of the surgical and anesthesia teams. Lipid emulsion should be promptly available whenever local anesthetics are employed, and its administration should commence promptly upon suspicion of LAST [39,40,41,42,43]. Simulation-based training in resuscitation techniques can enhance survival rates in patients experiencing LAST. Telemetry monitoring can enhance patient safety and facilitate the timely diagnosis of LAST.

### Clinical Implications

While this meta-analysis suggests that TAP block reduces pain and opioid consumption, TSA suggests the need for more high-quality and adequately powered RCTs to confirm this effect. Clinicians should interpret these results with caution. TAP block might still be beneficial, but its widespread use and adoption should be tempered until stronger confirmatory evidence emerges.

Given the low risks and complications associated with TAP blocks and their potential benefits, they may still be considered as part of multimodal analgesia, but clinicians should be aware that the evidence remains uncertain.

## 5. Conclusions

TAP block can lower pain intensity and reduce morphine consumption on the first post-surgical day in patients undergoing renal transplantation. Time to first analgesia was longer in the TAP block group. There was no significant difference between TAP block and control groups in the rate of postoperative nausea and vomiting. The subgroup analysis showed that pain reduction is statistically significant in the subgroup of donors, but the benefits reported are minimum and certainly not clinically relevant. Larger, well-powered RCTs are warranted to confirm these results and evaluate the effect of TAP block in the subgroup of recipients.

## Figures and Tables

**Table 1:** Study characteristics and description of included studies. Abbreviations: C, Control; ESKD, end-stage kidney disease; inj., injection; IV, intravenous(ly); LA, local anesthesia; N, number; NRS, Numeric Rating Scale; PCA, patient-controlled analgesia; PO, postoperative(ly); RCT, randomized controlled trial; VAS, Visual Analogue Scale.

First Author, Year	Country, Study Design	Study Goals	Age (TAP/C)	N (TAP/C)	Local Anesthetics, Volume and Concentration, Adjuvants	Postoperative Analgesia
Abdelsalam, 2014, [13]	Saudi Arabia, RCT	To assess the analgesic effectiveness of the pre-incisional TAP block in renal transplant patients	38 ± 13.41 34.4 ± 13.15	30/30	TAP: 20 mL bupivacaine 0.5% No TAP: 20 mL saline	PACU: IV morphine 2 mg bolus every 5 min at VAS ≥ 5, max 10 mg Post-PACU: IV paracetamol 1 mg 6 h, PCA morphine 1 mg, lockout 6 min, max 25 mg in 4 h
Boonyapalanant, 2022, [14]	Thailand, RCT	To compare the effectiveness of local infiltration and TAP block in living donor and cadaveric renal transplantation in patients with ESKD	44.9 ± 9.7 48.4 ± 9.7	22/23	TAP: 0.25% bupivacaine 20 mL LA: 0.25% bupivacaine 20 mL	Acetaminophen 0.5 g orally every 6 h At NRS > 3: 1 mg morphine IV
Can, 2015, [5]	Turkey, RCT	To evaluate TAP block on pain intensity (VAS) and total morphine requirement	51.5 ± 10.9 46.6 ± 11.5	25/22	TAP: 20 mL 0.5% bupivacaine C: 20 mL saline	IV PCA: morphine 1 mg, lockout 10 min + regular acetaminophen 1 g every 6 h
Freir, 2012, [4]	Ireland, RCT	To evaluate effectiveness of TAP block in deceased donation kidney recipients with ESKD	51.6 ± 13.2 44.6 ± 13.0	32/33	TAP: 20 mL levobupivacaine 0.375% C: 20 mL 0.9% saline	IV-PCA: morphine sulfate 2 mg every 5 min until VAS ≤ 3. Rest of 24 h: morphine 1 mg bolus, lockout 7 min, max 30 mg in 4 h + acetaminophen 1 g orally every 6 h
Gulyam Kuruba, 2014, [1]	UK, RCT	To assess effect of TAP on morphine use on first post-surgical day following kidney transplantation	51.3 ± 14.5 50.1 ± 13.5	24/27	TAP: 20 mL levobupivacaine 0.5% C: 20 mL saline 0.9%	IV paracetamol 1 g every 6 h IV PCA: morphine 0.5 mg every 10 min on demand
Hosgood, 2012, [6]	UK, RCT	To evaluate safety and efficacy of TAP block in laparoscopic living donor nephrectomy	52 ± 10 47 ± 9	24/22	TAP: 20 mL bupivacaine 0.375% C: 20 mL saline 0.9%	IV-PCA: morphine 1 mg bolus, lockout 5 min Then, up to 4 times a day: Tramadol 50–100 mg orally, paracetamol 1000 mg
Ibrahim, 2017, [15]	Saudi Arabia, RCT	To compare opioid use and pain scores between TAP and control groups of kidney recipients	45 ± 6.1 42 ± 5.2	25/25	TAP: 30 mL bupivacaine 0.25% Control: saline	Paracetamol 1 g IV every 6 h + IV-PCA fentanyl 10 μg/mL, doce 1 mL, lockout 6 min, not continuous.
Parikh, 2013, [16]	India, RCT	To examine analgesic effect of TAP in retroperitoneoscopic donor nephrectomy	45 ± 9.6 42 ± 7	30/30	TAP: 25 mL bupivacaine 0.375% C: 25 mL normal saline	Rescue: inj. Ondansetron 0.15 mg/kg IV, then 1 mg/kg inj. Tramadol at VAS > 3
Parikh, 2015, [17]	India, RCT	To examine analgesic effects of continuous TAP in open kidney transplant recipients	37.35 ± 11.17 44.47 ± 12.78	20/20	TAP: 1 mg/kg bupivacaine 0.25% C: normal saline infusion	Rescue: Pentazocine 0.3 mg/kg at VAS > 3
Soltani Mohammadi, 2014, [3]	Iran, RCT	To assess pain intensity and opioid use on first day following renal transplantation	38.5 ± 10.1 39.7 ± 8.0	22/22	TAP: 15 mL bupivacaine 0.25% + 5 μg/mL epinephrine C: Saline	IV-PCA: 1 mg morphine every 10 min on demand until NRS = 3
Yang, 2020, [2]	China, RCT	To compare effects of TAP, TAP with dexmedetomidine, and morphine-only anesthesia in open cadaveric kidney transplant recipients	41.6 ± 7.6 37.5 ± 10.3	19/20	TAP: 30 mL 0.33% ropivacaine C: morphine PO IV-PCA	All groups: IV-PCA (morphine 1 mg/mL; no background infusion, bolus 2 mg; lockout 8 min for 24 h) + boluses of 2 mg morphine allowed upon request

**Table 2:** Cochrane risk of bias 2. “+” Low risk of bias. “?” Some concerns.

	Bias Arising from the Randomization Process	Bias Arising from Deviations from the Intended Interventions	Bias Arising from Missing Outcome Data	Bias Arising from the Measurement of the Outcome	Bias Arising from the Selection of the Reported Results	Overall Risk of Bias
Abdelsalam 2014, [13]	+	+	+	+	?	?
Boonyapalanant 2022, [14]	+	+	+	+	+	+
Can 2015, [5]	?	+	+	+	+	+
Freir 2012, [4]	+	+	+	+	+	+
Gulyam Kuruba 2014, [1]	+	+	+	+	+	+
Hosgood 2012, [6]	+	+	+	+	+	+
Ibrahim 2017, [15]	+	+	+	+	+	+
Parikh 2013, [16]	+	+	+	+	+	+
Parikh 2015, [17]	?	+	+	+	+	?
Soltani Mohammadi 2014, [3]	+	+	+	+	+	+
Yang 2020, [2]	+	+	+	+	+	+

**Table 3:** Summary of findings.

Outcomes	Mean Difference [95% CI]	Risk Ratio [95% CI]	Number of Participants (Studies)	Certainty of the Evidence (GRADE)
Pain intensity during first 24 h PO	−0.65 [−0.88, −0.42]	-	547 (11)	⊕⊕◯◯ Low ^a^
Morphine requirements within 24 h PO (mg)	−4.82 [−7.87, −1.77]	-	189 (4)	⊕◯◯◯ Very low ^b^
Cumulative morphine requirements in 24 h PO (mg)	−14.13 [−23.64, −4.63]	-	369 (7)	⊕◯◯◯ Very low ^b^
Time to first analgesia (hours)	5.92 [3.63, 8.22]	-	138 (3)	⊕⊕◯◯ Low ^a^
Postoperative nausea and vomiting within 24 h PO	-	0.91 [0.49, 1.71]	306 (6)	⊕◯◯◯ Very low ^b^

^a^ due to inconsistency. ^b^ due to inconsistency and imprecision.

## Data Availability

The data presented in this study are available on request from the corresponding author.
